# Experimental Procedure for Warm Spinning of Cast Aluminum Components

**DOI:** 10.3791/55061

**Published:** 2017-02-01

**Authors:** Matthew J. Roy, Daan M. Maijer

**Affiliations:** ^1^School of Mechanical, Aerospace, and Civil Engineering, University of Manchester; ^2^Materials Engineering, University of British Columbia

**Keywords:** Engineering, Issue 120, flow forming, metal spinning, shear forming, A356, aluminum, near-net shape manufacturing

## Abstract

High performance, cast aluminum automotive wheels are increasingly being incrementally formed via flow forming/metal spinning at elevated temperatures to improve material properties. With a wide array of processing parameters which can affect both the shape attained and resulting material properties, this type of processing is notoriously difficult to commission. A simplified, light-duty version of the process has been designed and implemented for full-size automotive wheels. The apparatus is intended to assist in understanding the deformation mechanisms and the material response to this type of processing. An experimental protocol has been developed to prepare for, and subsequently perform forming trials and is described for as-cast A356 wheel blanks. The thermal profile attained, along with instrumentation details are provided. Similitude with full-scale forming operations which impart significantly more deformation at faster rates is discussed.

**Figure:**
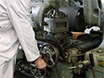


## Introduction

One of the more challenging metal forming operations currently being practiced in the aerospace and transportation sectors is metal spinning, including derivatives such as shear forming and flow forming^1^,^2^. In this process, an axisymmetric workpiece is placed on a mandrel representing the final desired shape, and spun into contact with one or more impinging rollers. The workpiece being compressed between the roller and mandrel then plastically deforms, with a diverse response including combined bending, thinning and axial elongation. In a material which has limited ductility or is otherwise difficult to form, this is sometimes carried out at elevated temperature to decrease flow stress and increase ductility.

From a processing standpoint, there are a wide range of parameters which can dictate the shape and properties of the manufactured component. Numerous studies have focused on statistical techniques for optimizing various parameters^3^,^4^,^5^. Variables include tooling geometry, such as the shape of the tool and mandrel; forming speeds including both mandrel rotation rate and tooling feed rates; as well as material properties. When elevated temperatures are required, practitioners need to assess the minimum temperature required while still retaining a sound product.

Cast aluminum alloys are employed in a wide variety of automotive and aerospace applications, with alloy A356 used in automotive wheels. However, this alloy is not suitable for forming at room temperature^6^,^7^ owing to its limited ductility and must be formed at elevated temperatures. This introduces a host of processing complexity, principally in controlling temperature. As this material's properties change significantly with temperature^8^, it is particularly important to perform instrumented trials in which thermal conditions can be kept to within a reasonable processing window and be monitored. Detailed data on the thermomechanical behavior of as-cast A356 ranging from ambient temperature to 500 °C over a wide range of strain rates can be reviewed elsewhere.^9^

In order to support development and optimization of flow forming operations for wheel manufacturing, custom forming equipment has been developed at the Department of Materials Engineering at the University of British Columbia (**Figure 1**). This apparatus has been built primarily from a manual, belt-driven capstan lathe with a total output of 22 kW, and a propane torch heating system with a peak output of 82 kW (**Figure 2**). A mandrel with embedded thermocouples along with a rigid roller assembly (**Figure 3**) has been installed, which is capable of forming workpieces up to 330 mm in diameter. The mandrel has a manually activated clamping system which is able to account for large changes in workpiece diameter occurring during processing (**Figure 4**). A battery operated Data Acquisition (DAQ) system containing a miniature wireless computer capable of monitoring the temperature of the mandrel during forming and the blank for characterizing heating has been installed on the quill of the lathe. While other flow forming processes have been synthesized using adapted lathes^4^,^10^, the present apparatus is the first to embody *in situ* heating and thermal data acquisition.

A processing protocol for industrially-scaled forming operations has been developed to provide indicative processing conditions. Described subsequently, this protocol consists of tooling and workpiece preparation, forming practice, concluding with end of forming trial operations.


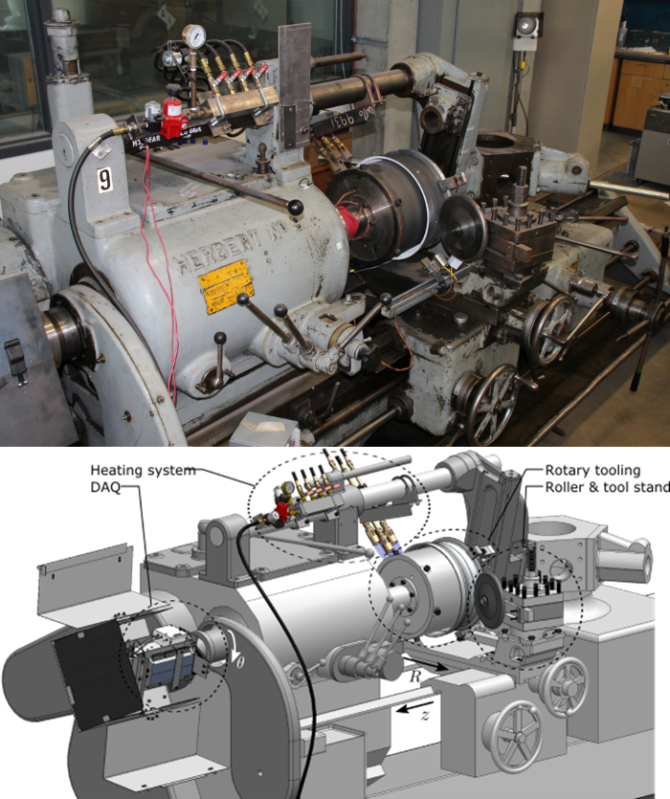
**Figure 1:Experimental apparatus overview.** Principle components which have been added to a modified capstan lathe for forming at elevated temperatures. Photograph of equipment (top) and main working directions and components labelled on a computer-aided design depiction (bottom). Please click here to view a larger version of this figure.


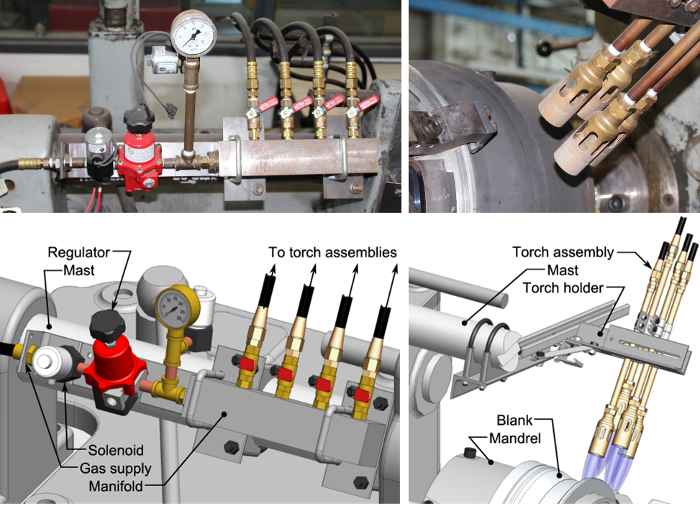
**Figure 2: Heating system detail.** A propane heating system with four discrete burners (top and bottom right) actuated from a central manifold containing a gas control solenoid (top and bottom left). Gas pressure and a discrete flow rate to each of the burners is possible, along with placement along the blank to conform to different geometries. Please click here to view a larger version of this figure.


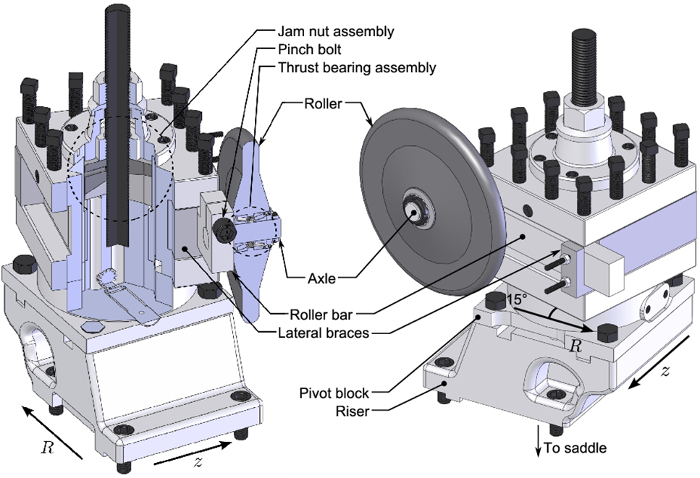
**Figure 3:Roller stand assembly detail.** The original tool holder on for the lathe has been adapted to hold a roller at arbitrary angles relative to the turning axis of the mandrel via a jam nut assembly. Please click here to view a larger version of this figure.


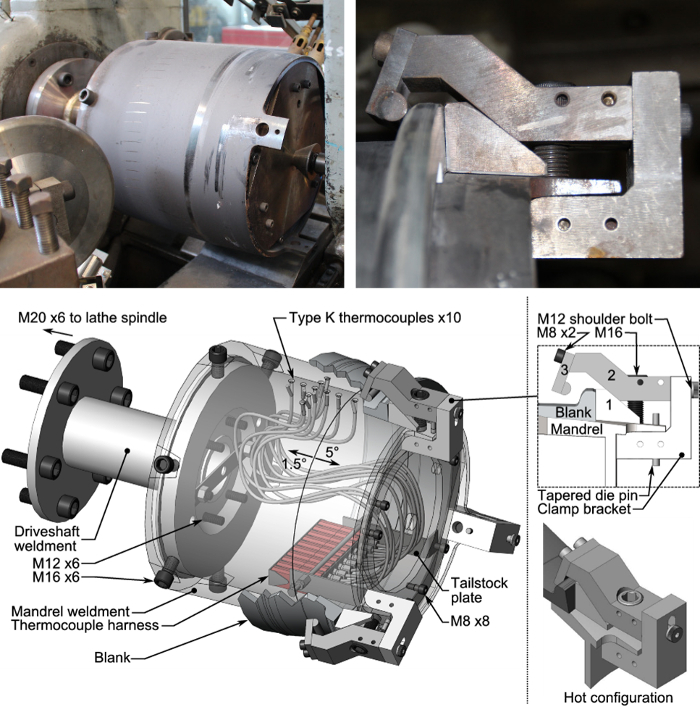
**Figure 4:Instrumented mandrel and clamp system overview.** The rotary tooling has been designed to bolt directly to the lathe spindle, which is in turn supported by a live center on the tailstock (top and bottom left). Clamp assembly/operation is also depicted (top and bottom right). Please click here to view a larger version of this figure.

## Protocol

### 1. Workpiece Preparation for Forming Trials

1. Acquire as-cast workpieces machined to the mandrel size such that the inner diameter runout is 0.2 mm, while the outer diameter retains as much cast surface as possible. NOTE: If blanks are drawn from full-size wheel castings, machining operations are required to remove all hub and spoke portions, while providing features which can be employed to clamp the workpiece to the mandrel. This includes removal of the in-board flange.
2. Pre-heat a coffin furnace able to receive the entire workpiece to 135 °C, clean the workpiece with degreaser and place in furnace for an hour to prepare for thermal barrier coating application.
3. Rapidly remove the workpiece from the furnace, and place on a coating jig. Using an automotive-type paint sprayer, apply a thin layer of thermal barrier die coating to the inner diameter. NOTE: This coating will provide lubrication and reduce heat transfer to the mandrel during forming operations.

### 2. Tooling Preparation

1. Wipe down the mandrel surface with a damp cloth. Ensure that the mandrel has a total rotational runout of < 0.5 mm using a dial gauge indicator along the forming length. Assess this with a live tooling center engaged on the tailstock plate. Using a torque wrench, ensure that all fasteners aside from those on the clamp assemblies are tightened to specified torque values for Grade 12.9 bolts (in Nm: M8 - 40, M12 - 135, M16 - 340).
2. Start the pre-heating system by first powering the gas supply solenoid, and then igniting the torches with a flint spark lighter. Run the pre-heating system for 10 min to expel any condensate collected in the torches/hoses. Extinguish by deactivating the gas supply solenoid.
3. Remove any loose/oxidized coating layer on the mandrel with dry 600/P1200 grit silicon carbide paper while turning the mandrel at 20 rotations per minute (rpm).
4. Power the on-board data acquisition module, and run the pre-heating system until the thermocouples embedded in the mandrel surface read 200 °C with the live center engaged.
5. Using an automotive-type paint sprayer, lightly coat the mandrel surface with a water-based forging lubricant and allow the rotary tooling to cool to ambient temperature with the live tooling center engaged.
6. Loosen the jam nut assembly on the roller stand (**Figure 3**) with a wrench. Set the approach or attack angle on the roller assembly using a toolmaker's protractor, and tighten both internal and external nuts (M35 - 750 Nm).
7. Assemble the 3 clamp assemblies (**Figure 4**) by first engaging the M12 shoulder bolt to connect element 2 to the clamp bracket. Inspect for any thermal distortion which will prevent element 2 in **Figure 4** from smoothly running against the clamp bracket. Ensure that they move freely, lightly sanding the contact surfaces with dry 320/P400 grit silicon carbide paper. Apply a thin layer of high temperature molybdenum-based lubricant with a cloth as needed.

### 3. Forming Operations

1. Move the roller tool stand completely away from the mandrel towards the spindle, move the tailstock and center to be clear of the mandrel. Manually slide the workpiece onto the mandrel ensuring even engagement. NOTE: As the blanks are nominally axisymmetric, there is no preferred orientation.
2. Assemble the clamps onto the mandrel by engaging the tapered die pins and hand tightening M16 bolts running through the mandrel into the clamp blocks. Ensure that there is even pressure being applied by rotating and manually tightening, followed by a pneumatic impact wrench set to 50 Nm.
3. Start the heating system and immediately start the mandrel rotating at 20 rpm. Keep applying heat until clamps loosen. For the process considered, this is approximately 3 min. NOTE: This time will be slightly different for each workpiece due to subtle differences in workpiece/mandrel fitment.
4. Extinguish the heating system and stop the rotation of the mandrel such that the first clamp is accessible with an impact wrench. Within 30 s, tighten all clamps with an impact or manual wrench and record the surface temperature of the workpiece in 3 locations along the length of the forming region with a reed-type thermocouple probe.
5. Repeat step 3.4 until the workpiece is at an appropriate forming temperature; at a minimum, 350 °C for A356. Perform a final tightening of the clamps with an impact wrench set to 200 Nm.
6. Move the roller axially and radially (approx. 2-5 mm from workpiece surface) into position for forming, and perform one last clamp tightening (*i.e.* step 3.4).
7. With the heating system on, increase the rotation rate of the lathe to the intended forming speed, engage the roller to a pre-set depth into the workpiece, and activate the screw-cutting feed to move the roller axially along the length of the workpiece. NOTE: For the present geometry, reasonable results were attained at 281 rpm with an axial movement of 0.21 mm/revolution.
8. Repeat Step 3.7 as required to increase levels of deformation. After each forming pass, ensure that the temperature does not drop below the optimal forming temperature by stopping the mandrel and using the same reed-type thermocouple probe as employed in step 3.4. If the optimal forming temperature has dropped, repeat steps 3.4 and 3.5 to reheat. NOTE: Reheating can be employed, however at the expense of potentially reaching the extent of the clamp system's ability to restrain the workpiece.

### 4. Post Forming Operations

1. Once the desired level of deformation has been obtained, stop the heating system, and undo all clamps, and disengage the tailstock to obtain clearance for workpiece removal.
2. Gently tap the workpiece with a piece of brass to separate from the mandrel. If this proves to be ineffective, re-engage the heating system and rotate the mandrel at 20 rpm gently tapping until the blank separates.
3. Using an appropriate manipulation tool such as tongs or heavily insulated gloves, either quench the workpiece in water at 60 °C to prevent further ageing, or leave to air cool to minimize residual stress/distortion.

## Representative Results

As-cast aluminum A356 workpieces were formed according to the method described in this paper. The workpieces were obtained from as-cast wheels from a North American wheel manufacturer employing the low-pressure die casting process. One workpiece instrumented with thermocouples was not formed, but underwent the pre-heating cycle (Protocol Section 3, steps 3.3-3.5) to capture the distribution of temperature across the surface of the blank during this aspect of the process. This response is shown in **Figure 5**. A further 3 samples were deformed to various levels, including one which received two forming passes for a high level of deformation. The first two samples and the first pass performed on the latter sample served to straighten the workpiece with little demonstrable change in wall thickness. The latter sample peak wall thickness reduction was approximately 10%, the majority of which was achieved in the second pass. Cross-sections and microstructure of the as-cast blank and those obtained in multi-pass sample are shown in **Figure 6**. Here, the as-cast microstructure is shown to significantly be refined by the process with dendritic features barely discernable. The interdendritic eutectic is broken up by the deformation imposed, creating a much more homogenous microstructure than in the as-cast state. This improves the overall ductility as well as fatigue and fracture properties of the component. The authors have previously described more details of workpiece geometry, specific cross-sectional changes in wall thickness, defects observed, and dimensional variation in microstructure on the full set of samples^8^,^13^.


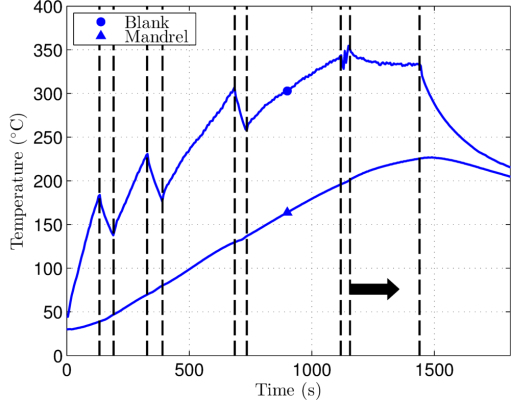
**Figure 5:Typical temperature profile of mandrel and blank.** A representative transient thermal response of the blank and mandrel obtained with the heating system. Vertical dashed lines indicate where clamps were tightened during the preheating steps, and the black arrow depicts forming. The last vertical line shows where the heating system was turned off whilst the system cooled.


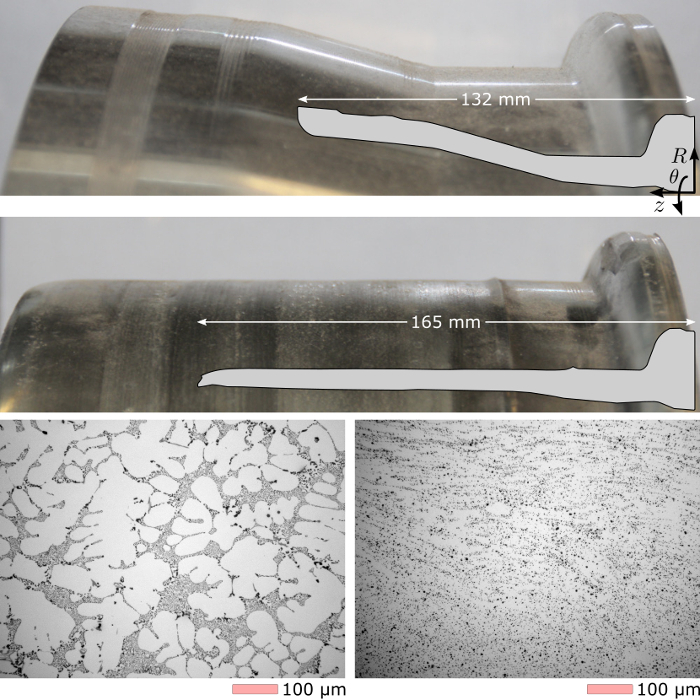
**Figure 6: As-cast and formed result.** The as-received, as-cast blank surface and geometry having a minimum inner diameter of 330 mm (top) was deformed in two passes to provide the result shown (middle). The as-cast dendritic microstructure (bottom left) is visibly modified by the forming operation and a subsequent T6 heat treatment (bottom right) as observed with optical microscopy^8^,^13^. Please click here to view a larger version of this figure.

## Discussion

The representative results shown above highlight that the protocol and equipment employed is capable of forming cast aluminum at elevated temperatures, and has provided a platform to determine a processing window for flow forming of wheels. The technique demonstrated can be used to explore aspects of forming envelopes, including how both formed and unformed material responds to heat treatment^8^. However, there is room for improvement with the current processing protocol with this apparatus.

Regarding further instrumentation, which would accelerate process model development, the inclusion of machine-tool dynamometer and tribometers^11^,^12^ to measure forming loads and friction factors on the roller would provide important information about the process conditions. This is a widely employed instrumentation technique for orthogonal machining studies, and could be readily implemented on the current machine. This additional instrumentation would provide useful data to accurately validate of modelling efforts^13^,^14^ and support the increasing industrial interest in this process. In order to effectively capture the evolution of temperature of the blank during processing, a non-contact measurement technique is desirable. However, common infrared-based techniques are hampered by aluminum's low emissivity and how the surface changes during processing. This is the principal reason why an instrumented, commissioning blank was employed to capture the typical thermal response achieved with the protocol described, and served to populate a baseline heat transfer analysis to relate mandrel surface temperature to the workpiece.

As it is largely a manual forming process for a material which is sensitive to time at temperature, some inconsistencies between run to run are to be expected. Aluminum alloys have microstructures that are highly sensitive to temperatures above 100°C due to ageing mechanisms. Therefore, the most critical steps within the protocol are 1.2 and 3.3-3.7, where the blank is at elevated temperatures. Tightening and re-seating the clamps must be conducted as quickly as possible to maintain repeatability between forming operations.

The *in situ* workpiece heating employed during the pre-heating step is quite inefficient and could be improved via radiative heating. The overall processing speeds in terms of mandrel and tool movements that can be attained are somewhat limited by the capabilities of the lathe employed. Higher forming speeds require a more rigid frame with a higher load capacity, particularly if the forming of a stronger material were to be attempted. Workpiece clamping and release could be improved with the addition of hydraulic or pneumatic actuation. As heat transfer from the blank to the mandrel is largely a function of the pressure imposed by the workpiece onto the mandrel, this addition could also improve a model-based approach to ascertain the workpiece temperature during forming with the existing system.

The apparatus and procedure described has shown that forming loads for this material under these conditions approaches those for standard turning operations, and remains a very cost effective process by which to perform manufacturing trials. Research into different manufacturing routes and formability can be performed away from commercial forming equipment, which is exceedingly expensive to operate. With the apparatus and protocol described, processing parameters can be investigated prior to constructing larger scale, higher throughput equipment, and to the authors' knowledge, is a unique approach.

As the protocol developed has only been applied to one specific variant of cast aluminum alloy, there is a multitude of other aluminum foundry alloys which could be investigated for a variety of applications beyond automotive wheels. As these alloys have approximately similar processing windows from a temperature perspective, the protocol developed can be readily adapted.

## Disclosures

The authors have nothing to disclose.
